# *Candida albicans ISW2* Regulates Chlamydospore Suspensor Cell Formation and Virulence *In Vivo* in a Mouse Model of Disseminated Candidiasis

**DOI:** 10.1371/journal.pone.0164449

**Published:** 2016-10-11

**Authors:** Dhammika H. M. L. P. Navarathna, Ruvini U. Pathirana, Michail S. Lionakis, Kenneth W. Nickerson, David D. Roberts

**Affiliations:** 1 Laboratory of Pathology, Center for Cancer Research, National Cancer Institute, National Institutes of Health, Bethesda, Maryland, United States of America; 2 School of Biological Sciences, University of Nebraska-Lincoln, Lincoln, Nebraska, United States of America; 3 Fungal Pathogenesis Unit, Laboratory of Clinical Infectious Diseases, National Institute of Allergy and Infectious Diseases, National Institutes of Health, Bethesda, Maryland, United States of America; Universite de Nice - CNRS, FRANCE

## Abstract

Formation of chlamydospores by *Candida albicans* was an established medical diagnostic test to confirm candidiasis before the molecular era. However, the functional role and pathological relevance of this *in vitro* morphological transition to pathogenesis *in vivo* remain unclear. We compared the physical properties of *in vitro*-induced chlamydospores with those of large *C*. *albicans* cells purified by density gradient centrifugation from *Candida*-infected mouse kidneys. The morphological and physical properties of these cells in kidneys of mice infected intravenously with wild type *C*. *albicans* confirmed that chlamydospores can form in infected kidneys. A previously reported chlamydospore-null *Δisw2/Δisw2* mutant was used to investigate its role in virulence and chlamydospore induction. Virulence of the *Δisw2/Δisw2* mutant strain was reduced 3.4-fold compared to wild type *C*. *albicans* or the *ISW2* reconstituted strain. Altered host inflammatory reactions to the null mutant further indicate that *ISW2* is a virulence factor in *C*. *albicans*. *ISW2* deletion abolished chlamydospore formation within infected mouse kidneys, whereas the reconstituted strain restored chlamydospore formation in kidneys. Under chlamydospore inducing conditions *in vitro*, deletion of *ISW2* significantly delayed chlamydospore formation, and those late induced chlamydospores lacked associated suspensor cells while attaching laterally to hyphae via novel spore-hypha septa. Our findings establish the induction of chlamydospores by *C*. *albicans* during mouse kidney colonization. Our results indicate that *ISW2* is not strictly required for chlamydospores formation but is necessary for suspensor cell formation. The importance of *ISW2* in chlamydospore morphogenesis and virulence may lead to additional insights into morphological differentiation and pathogenesis of *C*. *albicans* in the host microenvironment.

## Introduction

*Candida albicans* is a commensal yeast fungus that is part of the human gastrointestinal and genitourinary tract microbiota. It has emerged as a significant opportunistic pathogen in the growing population of immunocompromised patients, where it causes considerable morbidity, mortality, and health care costs [1–4]. *C*. *albicans* is now the leading cause of nosocomial bloodstream infection in the USA [5]. Disseminated candidiasis is highly prevalent among immunocompromised cancer patients undergoing chemotherapy [6, 7]. Approximately $1 billion is spent annually to manage disseminated candidiasis, which has ~40% mortality rate irrespective of treatment with currently available antifungal drugs [8]. The unique morphological plasticity of this pathogen allows *C*. *albicans* to switch between unicellular yeasts, pseudohyphae, and hyphal forms and contributes to virulence [9]. Under certain environmental conditions, *C*. *albicans* also differentiates into mating competent opaque cells [10], specifically regulated commensal state GUT (Gastrointestinally Induced Transition) cells [11], and thick-walled chlamydospores [12].

Chlamydospores, first described in 1877 by Grawitz et al. [13], are the least studied of these morphologic forms and are the only spore type made by *C*. *albicans*. Chlamydospores are large, spherical, thick-walled, and refractile cells that are usually 8±1 μm in diameter, although values from 7 to 12 μm have been reported in the literature. Chlamydospores are thought to be formed by rounding off the terminal filamentous hyphae where the basal hyphal compartment has differentiated into a specialized ‘suspensor cell’. Therefore, suspensor cells are considered as a necessary precursor for chlamydospore formation in which nuclear division occurs according to the chlamydospore developmental model described by Martin et al. [14]. Laboratory conditions that induce chlamydospore formation include nutritionally poor media containing complex sugars such as corn meal or rice extract, low temperatures (24–28°C), darkness, and microaerophilic growth under glass cover slips. Chlamydospores are non-meiotic, asexual spores. Except for the presence of a thick cell wall, they do not share most common characteristics of classical fungal spores. Their physiological status and role *in vivo* are still uncertain, and they have only been useful for diagnostic differentiation of *C*. *albicans* and *C*. *dubliniensis* from other species of *Candida* and other clinically important yeasts [12, 14–22]. So far, little is known about the genetic regulation of chlamydospore development in *Candida* species. *C*. *albicans EFG1* is the major transcriptional regulator required for chlamydospore formation [20] but it also regulates hyphal transitions. Filamentation is also a prerequisite for chlamydospore formation [14]. *NRG1* and *HOG1* signaling are also associated with chlamydospore production [15, 23]. A recent study established that the *Candida* cell wall proteins Csp1p and Csp2p are induced specifically in the process of chlamydospore production [24].

It is unlikely that chlamydospore formation would persist through evolution without a biological function. The presence of *C*. *albicans* chlamydospores *in vivo* during an infection has been reported in a few older clinical case reports [25–27], although these reports were only based on microscopy data. In tissue sections from our previously published animal model studies [28–38], we have consistently observed large spherical cells similar in size to chlamydospores which are present in the outer perimeter of *C*. *albicans* colonies in the mouse kidney cortex (unpublished observations). Our group has remained curious whether these structures are in fact true chlamydospores.

Mutant library screening by Nobile et al (2003) found three mutants, *Δisw2/Δisw2* (CJN16), *Δsch9/Δsch9* (CJN19), and *Δsuv3/Δsuv3* (CJN223) that lacked the ability to induce chlamydospores [19]. Based on our preliminary analysis of chlamydospore phenotypes in these mutants, *Δisw2/Δisw2* was chosen to study the role of *ISW2* (orf19.7401) in chlamydospore development and in pathogenesis of disseminated candidiasis. Its closest characterized ortholog, *S*. *cerevisiae* Isw2p, is an ATP-dependent chromatin remodeling factor that belongs to the highly conserved ISWI (Imitation Switch) protein family [39–42]. Being an essential factor for protein-DNA interactions on chromatin, *ISW2* also affects the regulation of transcription, recombination, and DNA repair, although the exact mechanisms remain to be elucidated [43]. Moreover, Tsukiyama et al [43] showed that the ISWI gene complexes interact with each other to maintain the chromatin structure, thus allowing survival of yeast cells under stress conditions. Due to the partial localization of Isw2p with microtubules, this protein is also crucial for the expression of early meiotic genes by enabling transcription factors for sporulation specific genes to access chromatin, which in turn initiates sporulation in *S*. *cerevisiae* [42]. Isw2p also exhibits a repressor activity during mitotic growth of yeast cells following recruitment by Ume6p, a transcription factor involved in both positive and negative regulation of a diverse set of yeast genes [39, 40]. Therefore, *ISW2* may regulate other functions in *C*. *albicans* in addition to chlamydospore development.

The previously reported *ISW2* null mutant, CJN16, is derived from an avirulent BWP17 parent [19]. Therefore, we constructed an *ISW2* deleted mutant in a prototrophic WT SC5314 *C*. *albicans* background to investigate the role of this gene in a mouse model of disseminated candidiasis. We report here that deletion of *ISW2* delays the onset of chlamydospore formation without having any discernible defects on cell filamentation or its ability to cope with oxidative stress. Notably, the chlamydospores formed *in vitro* by the *Δisw2/Δisw2* mutant are unique in that they form without characteristic suspensor cells and instead form laterally, being attached directly to the hyphae. We report the role of *ISW2* in *in vivo* chlamydospore induction and virulence using a mouse model of disseminated candidiasis. In addition, we confirm previous clinical reports of *in vivo* chlamydospore formation using mouse models.

## Materials and Methods

### Ethics Statement

Handling and care of mice was conducted in an AAALAC International accredited facility in compliance with the guidelines established by the Animal Care and Use Committee of the National Cancer Institute under approved protocol LP-022. Mice that reached approved humane endpoints were euthanized by CO2 inhalation.

### Strains, media and growth conditions

*C*. *albicans* strains SC5314 [44] and A72 (ATCC MYA-2430) [45] were obtained from the American Type Culture Collection, Rockville, MD. *C*. *dubliniensis* Wü284 strain was kindly provided by Dr. Joachim Morschhäuser from the University of Würzburg, Germany. The strains constructed for this study are listed in Table 1. For routine growth of *C*. *albicans* strains, YPD medium (10 g/l of yeast extract, 20 g/l of peptone and 20 g/l of glucose with or without 20 g/l of agar) was used. To induce chlamydospore production, *C*. *albicans* cells grown overnight were plated onto inducing corn meal agar (DIFCO, Detroit, MI or Fluka, Sigma-Aldrich) plates supplemented with 1% Tween 80 according to the Dalmau technique described below. For RNA isolation, corn meal broth medium was prepared by modifying commercially available corn meal agar medium as described previously [46]. Briefly, powdered corn meal agar was suspended in 10% excess of distilled water, continuously stirred overnight at 4°C, and filtered. Staib agar was prepared as described previously [47] using 50 g of *Guizotia abyssinica* plant seed extracted into 1 liter of distilled water and then supplemented with 1 g of glucose, 1 g of KH_2_PO_4_, 1 g of creatinine, and 15 g of agar.

**Table 1:** *C*. *albicans* strains used or constructed in this study.

Strain	Genotype	Reference
SC5314	Wild type (*ISW2/ISW2*)	[44]
DRL5^NR^	*Δisw2*::*SAT1-FLP/ISW2*	This study
DRL5	*Δisw2/ISW2*	This study
DRL6 ^NR^	*Δisw2/Δisw2*::*SAT1-FLP*	This study
DRL6	*Δisw2/Δisw2*	This study
DRL7 ^NR^	*Δisw2/Δisw2*::*ISW2*::*SAT1-FLP*	This study
DRL7	*Δisw2/Δisw2*::*ISW2*	This study
BWP17	*ura3*Δ::λ*imm434/ura3*Δ::λ*imm434 his1*::*hisG/his1*::*hisG arg4*::*hisG/arg4*::*hisG*	[80]
CJN16	*ura3*Δ::λ*imm434/ura3*Δ::λ*imm434 arg4*::*hisG his1*::*hisG*::*pHIS1 isw2*::Tn*7-UAU1 arg4*::*hisG his1*::*hisG isw2*::Tn*7-URA3*	[19]
CJN19	*ura3*Δ::λ*imm434/ura3*Δ::λ*imm434 arg4*::*hisG his1*::*hisG*::*pHIS1 sch9*::Tn*7-UAU1 arg4*::*hisG his1*::*hisG sch9*::Tn*7-URA3*	[19]
CJN223	*ura3*Δ::λ*imm434/ura3*Δ::λ*imm434 arg4*::*hisG his1*::*hisG*::*pHIS1 suv3*::Tn*7-UAU1 arg4*::*hisG his1*::*hisG suv3*::Tn*7-URA3*	[19]
DAY25	*ura3*Δ::λ*imm434/ura3*Δ::λ*imm434 arg4*::*hisG his1*::*hisG*::*pHIS1 rim101*::*ARG4 arg4*::*hisG his1*::*hisG rim101*::*URA3*	[19]
BH1P1	*BMH1*/*bmh1*Δ::*HIS1 his1*Δ/*his1*Δ *arg4*Δ::*ARG4*::*URA3*/*arg4*Δ	[64]
UdR142C	*bmh1*Δ::*HIS1/bmh1*Δ::*ARG4 ura3*Δ/*ura3*Δ::*URA3*::*bmh1R142C*	[65]

For mouse infections, *C*. *albicans* cells were grown overnight in 50 ml of yeast extract-peptone-dextrose (YPD) medium at 30°C aerobically. The *C*. *albicans* yeast cells were harvested by centrifugation at 4200 × *g* for 10 min, washed twice with 50 ml of sterile nonpyrogenic normal saline, and resuspended in 10 ml of sterile nonpyrogenic saline before cell quantification using a Petroff—Hausser counting chamber. The cell suspensions were adjusted to a final concentration of 1 × 10^7^ cells/ml for parenteral administration using nonpyrogenic sterile saline.

### Harvesting and purification of chlamydospores *in vitro*

Radiating *C*. *albicans* colonies on agar plates were removed using a sterile blade and suspended in 3M sodium thiocyanate in TE buffer. The *C*. *albicans* laden agar pieces were incubated for 10 min at 50°C with intermittent vortexing to solubilize the agar. After centrifugation at 4,000 rpm for 5 min, the *C*. *albicans* cell-hypha pellet was resuspended in PBS buffer, and chlamydospores were separated from suspensor cells by sonication (30 s sonication and 30 s resting cycles for 5 min on ice).

Chlamydospores were purified on linear sucrose density gradients [48] of 35–66% w/w (1.15–1.32 g/cc), which were prepared by layering successive 2 ml fractions of decreasing density in 15 ml Beckman polyallomer tubes, whereupon 1–2 ml of sample in 35% sucrose was layered on top. Centrifugation was carried out at 39000 rpm in a SW41 swinging bucket rotor for 12 hours at 10°C in a L70 Beckman ultracentrifuge.

### Staining and microscopy of purified chlamydospores

Images of purified chlamydospores, either unstained or stained with Calcofluor White were obtained using light (Olympus, CH3-TR45) and fluorescence (Olympus, BX51TRF) respectively. Images were processed with IPLab software (Scanalytics Inc., Fairfax, VA). For chitin staining, 10% v/v formaldehyde fixed chlamydospores were treated with 0.2 μg/ml Calcofluor White for 5 min in the dark and washed once with xylene. The low concentration of Calcofluor White was chosen to stain chlamydospores predominantly, rather than yeast or hyphae. Size measurements were made with an ocular micrometer and presented as the mean ± standard error of 150 cells.

### Construction of *Δisw2/Δisw2* mutant and its complementation

To knock out the *ISW2* gene, we followed the *SAT1* flipping strategy reported by Reuss et al, [49] and Sasse & Morschhäuser, [50] using WT *C*. *albicans* strain SC5314 [44]. The pSFS2A plasmid was kindly provided by Dr. Joachim Morschhäuser from the University of Würzburg, Germany. The unique restriction sites on the left (*ApaI*, *XhoI*) and right (*SacI*, *SacII*) borders of *SAT1* flipper cassette with nourseothricin resistant marker was used for the construction of the *ISW2* gene disruption cassette. The primers used in this study are listed in the Table in S1 File. In brief, the *ApaI-XhoI* fragment of the *C*. *albicans ISW2* (0.46 kb) upstream flanking sequence was amplified using primer pair, ISW2upleft and ISW2upright. A *SacII-SacI ISW2* downstream fragment (0.42 kb) was amplified with primer pair ISW2downleft and ISW2downright. The *ISW2* downstream and upstream fragment was cloned to generate pSFS2A*ISW2down* and then the plasmid with complete gene disruption cassette, pSFS2A*ISW2*, respectively. Transformation for gene knock out was done as we described previously [34, 37] with the *ApaI-SacI* fragment from pSFS2A*ISW2*. The transformants were selected on YPD plates containing 200 μg/ml nourseothricin (JenaBioscience, Germany). The nouseothricin resistance marker in single allele knocked out strain (DRL5^NR^) was excised by growing in yeast extract-peptone-maltose (YPM) liquid medium as described by Reuss et al. (2004) [49]. This strain, named DRL5, was then transformed again with the same *ApaI-SacI* fragment to make DRL6^NR^ and, following another FLP-mediated excision, generating the *ISW2* homozygous gene knock out mutant, designated as DRL6. The clones were analyzed by sequential Southern hybridization at each step for desired allelic displacement and replacement, using *EcoR1* digested genomic DNA of the transformants with the *ISW2* up and downstream fragments as probes.

The *ISW2* gene complementation cassette was constructed with the *ApaI-XhoI* fragment of the complete *C*. *albicans ISW2* sequence as well as 0.34 kb of upstream and 0.174 kb of downstream flanking sequences for *ISW2* (3746 bp), amplified using primer pair ISW2compleft and ISW2compright. This fragment was sub-cloned to the *ApaI-XhoI* site in pSFS2A*ISW2down* to generate *ISW2* complementation cassette p*ISW2COMP*. We inserted one copy of the gene back to *ISW2* locus to make the strain *Δisw2*::*ISW2/Δisw2* which was designated as DRL7. Southern hybridization and qRT-PCR analyses confirmed the reintegration of the gene at the correct locus and expression of the reintegrated gene, respectively.

### Kinetics of chlamydospore formation *in vitro*

To induce chlamydospore production, log phase cells and cells grown overnight on fresh YPD liquid medium were used as the inoculum because the growth phase was reported to have effects on chlamydospore formation rate [18]. To obtain log phase cells, an overnight YPD liquid culture of *C*. *albicans* was inoculated into fresh YPD liquid medium at a cell density of ca. 0.01 OD_600_ and incubated for 4–5 hours at 30°C in a 225 rpm rotatory shaker. For each cell type, volumes of 3 μl were used to inoculate either corn meal agar plates supplemented with 1% Tween 80 or Staib agar plates, following the Dalmau technique where cover slips were placed on top of the inoculum to create microaerophilic conditions. The plates were incubated at room temperature in the dark. The plates were examined over the next 6 weeks for *in situ* chlamydospores using an AMG EVOSfl Digital Inverted Microscope.

### Filamentous growth and cell cycle growth analysis

The ability to form filaments at 30 or 37°C was determined by incubating *C*. *albicans* cells in YPD liquid medium as well as under embedded growth conditions. The embedded assay was performed by the pour plate technique with an inoculum of 10^4^ cells/ml in Glucose-Proline-Phosphate (GPP) agar and grown at 37°C for 15 to 17 hours. The ability to form pseudohyphae was also observed by growing the cells for 4 hours at 37°C in liquid GPP media supplemented with 300 or 600 mM phosphate (1:1 KH_2_PO_4_:K_2_HPO_4_), as described in [51]. All growth patterns were observed *in situ* using AMG EVOSfl Digital Inverted Microscope.

Cell cycle progression was analyzed by nuclear staining with propidium iodide at each time point using a protocol modified from Zhang & Siede [52]. To initiate the cell cycle, G_1_ synchronized cells were obtained by a slight modification of a previous protocol [53]. In brief, YPD grown cells were inoculated at 2 × 10^8^ cells/ml into synthetic complete medium without glucose. This carbon deficient liquid medium contained 6.7 g/l of yeast nitrogen base without amino acids (Sigma Y0626), 1.92 g/l of yeast synthetic dropout media without uracil (Sigma Y1501), and 20 mg/l of uracil. Overnight grown cells were inoculated at a final cell density of 2 × 10^7^ cells/ml into fresh YPD liquid medium, grown at 30°C aerobically and then harvested after 0, 1, 2 and 3 hours. The harvested cells were washed once with deionized water, fixed in absolute ethanol for 1 hour, mixed with an aliquot of fresh absolute ethanol, and stored at 4°C until all the samples had been collected. To prepare for flow cytometry, the samples were vortexed extensively, centrifuged for 1 min at 14,000 × g, washed with 1 ml of water, and centrifuged again. The cell pellets were resuspended in 1 ml of 50 mM sodium citrate (pH 7.0) and transferred to FACS analysis tubes (BD Falcon 2054) and treated with 8 μl volumes of 10 mg/ml DNase-free RNase A for 1 hour at 50°C. Samples were then incubated with 25 μl of 10 mg/ml proteinase K for another hour. Finally, 1 ml of 20 μg/ml propidium iodide in 50 mM sodium citrate (pH 7.0) was added in the dark. All samples were briefly sonicated just before analysis on a BD FACSCanto^™^ II with FACSDiva version 6.1.1 software. Actual cell numbers were statistically analyzed using ANOVA on GraphPad Prism software.

### Mouse infection with *C*. *albicans*

Outbred 6–8 week old (18–20 g) BALB/c female mice obtained from the NCI Frederick mouse breeding facility were randomly allocated to groups of five animals and housed and cared with *ad libitum* access to filtered water and standard mouse chow. For the survival study, WT (SC5314), DRL6 (*Δisw2/Δisw2)*, and DRL7 (*Δisw2*::*ISW2/Δisw2)* strains were inoculated into three groups of 15 mice with a control group getting only sterile non pyrogenic saline. Each group was inoculated intravenously in the lateral caudal tail vein using a 27 gauge needle with a volume of 0.1 ml containing 10^6^ *C*. *albicans* cells of each respective strain [28, 30, 31]. Clinical signs of illness in each mouse were evaluated three times daily, and mice that displayed humane endpoints including arched back posture, sunken eyes, ruffled hair, or dehydration were euthanized immediately by CO_2_ inhalation and processed for complete necropsy and collection of tissues for histopathological examination. To examine basic host immune responses, two groups of mice were infected with the WT SC5314 and DRL6 strains, and euthanized at 2 days post-inoculation (PI) for organs and sera collection. Five mice were inoculated with the WT SC5314, five were inoculated with DRL6 strain, and five control mice received 0.1 ml saline i.v with no fungal challenge. Sera separated from the blood collected from individual mice were stored at -80°C until analysis. Kidneys of infected mice consisting of 5 mice per group infected with WT SC5314, DRL6, or DRL7 strains were stored at -80°C for subsequent use to extract total RNA for analysis of chlamydospore-specific gene expression at day 3 post-infection.

### Necropsy and histopathology

Immediately after euthanasia, macroscopic changes were recorded, and the brain, heart, lungs, liver, spleen, and right kidneys were immersed in buffered 10% formalin, processed for paraffin embedding, sectioned at 5 μm, and stained with haematoxylin and eosin (H&E). Grocott's modification of Gomori's methenamine-silver (GMS) stain was used for detection of fungi *in situ* [35, 37]. Histopathology images from sections of formalin-fixed and paraffin-embedded tissues stained with GMS or H&E were obtained using a light microscope (Olympus BX51) fitted with a digital camera (Nikon DXM1200F) and ScanScope XT digital scanner (Aperio). Images were processed with Adobe Photoshop and Aperio ImageScope v11.1.2.760 (Aperio).

### Determination of serum cytokines and chemokines

Murine serum was collected from sacrificed mice at 2 days PI following infection with *C*. *albicans* WT and mutant strains. A Luminex-bead array (Mouse cytokine/Chemokine Milliplex MAP Kit, catalog no MPXMCYTO-70K, Millipore, Billerica, MA) was used to detect the cytokines IL-6, IL-10 IL-17, TNF-α, IL-1β, GM-CSF and chemokines MCP1, MIP1-α and RANTES according to the manufacturer's specifications.

### Statistical analysis

The probability of survival as a function of time was determined by the Kaplan-Meier method, and significance was determined by the log-rank (Mantel-Cox) test and Jehan-Breslow-Wilcoxon test using GraphPad Prism software. Serum cytokine expression patterns among all treatment groups at 2 days PI were analyzed by two-way ANOVA with the post Bonferroni comparison test. Three to four randomly selected mice from each group were euthanized at each time point for longitudinal comparisons. Data were analyzed for significant differences by comparing means of each triplicate reading at various time points assuming that the cytokine expression levels within each group of mice were normally distributed.

### Expression of chlamydospore specific markers *in vitro* and *in vivo*

For *in vivo* gene expression analysis, total RNA was extracted from kidneys infected with *C*. *albicans* WT, DLR6, and DLR7 strains. *In vitro* gene expression analysis was performed in the same strains grown in corn meal broth and YPD broth at different time points up to 5 days using a phenol extraction method as described [54], and the absence of genomic DNA was confirmed by a forward PCR primer within the *ACT1* intron (P26) and a reverse primer within the distal exon (PN90) as described previously [55] and/or DNAse treatment using TURBO DNA-free ^™^ Kit (Invitrogen). One hundred ng of total RNA were used to prepare first strand cDNA using SuperScript^®^ III First-Strand Synthesis SuperMix for qRT-PCR (Invitrogen^™^) according to the manufacturer’s recommendation using oligo(dT) primers. Quantitative PCR was conducted using iQ^™^ SYBR^®^ Green Supermix in a Biorad CFX Connect^®^ real-time PCR detection system. Each cDNA preparation was normalized using *CaCDC36* as an internal control. The primers used in this study are listed in the Table in S1 File. Quantitative RT-PCR data were normalized in two steps as described previously [34] and analyzed using two-way ANOVA with the post Bonferroni comparison test.

## Results

### Chlamydospore-like structures in mouse kidneys infected with *C*. *albicans*

**Fig 1:**
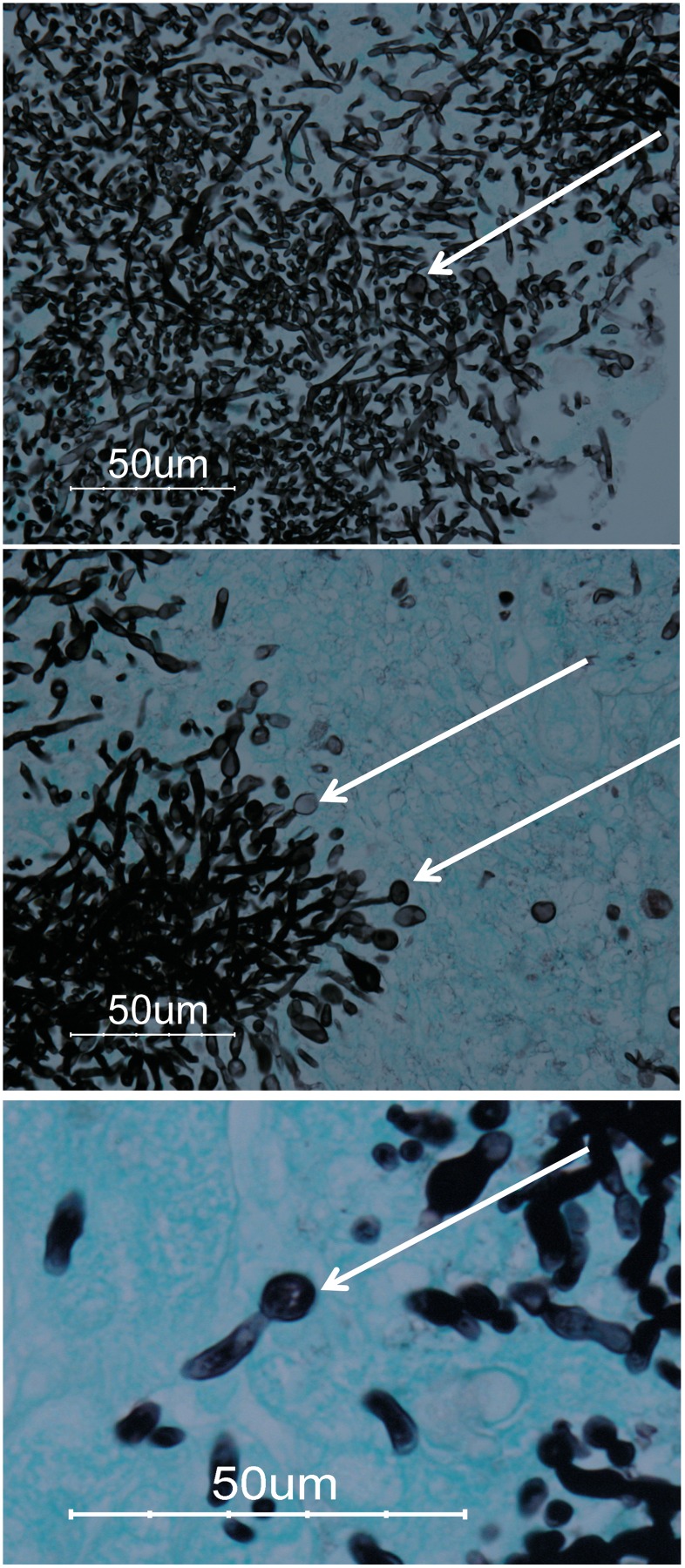
Chlamydospore-like structures are observed in *C*. *albicans*-infected mouse kidneys at day 3 post-inoculation. Top panel: A representative section from infected kidney cortex stained using GMS three days PI with *C*. *albicans*. Chlamydospore-like structures are indicated by the arrows. Middle panel: Periphery of a resolving *Candida*-infected lesion shows isolated chlamydospore-like structures (arrows). Bottom panel: Higher magnification of the same histopathology sections shows spherical fungal spores with a diameter of 8 μm.

Kidneys are the primary colonization organ in the mouse model of disseminated candidiasis [30, 31, 56, 57]. The mouse immune system can clear the initial *C*. *albicans* infection from other organs, so the outcome of the infection depends on how fungal colonization progresses in the kidneys [58]. Yeast cells escape from nephrons and settle in peri-glomerular regions of the kidney as early as 2 to 6 hours PI [34]. Initially, the yeast cells grow as micro-colonies composed of mixtures of yeasts and filaments, which subsequently overwhelm the kidney cortex as early as 2 days PI [30, 34]. The host response consists of strong monocyte/macrophage and neutrophil infiltration together with high levels of TNF-α and IL-6 and other pro-inflammatory cytokines, which creates a local inflammatory response, leading to tissue injury and necrosis, kidney malfunction, and the formation of granulation tissues around the *C*. *albicans* colonies starting at 3 to 7 days PI [34, 37]. At this stage we observed large spherical cells ca. 6–8 μm in size in histopathological sections stained with GMS (Fig 1). These spherical cells were limited in number and were mainly visualized in the periphery of the resolving lesions caused by extensive fungal colonization (Fig 1, middle panel). We frequently observed these large spherical cells in the kidney cortexes of mice from day 3 PI onwards (Fig 1), and we have observed them in most of our previous studies [28–38] with both WT *C*. *albicans* A72 and SC5314 in the mouse model for disseminated candidiasis (unpublished observations).

### *In vitro* and *in vivo* characterization of chlamydospores

**Fig 2:**
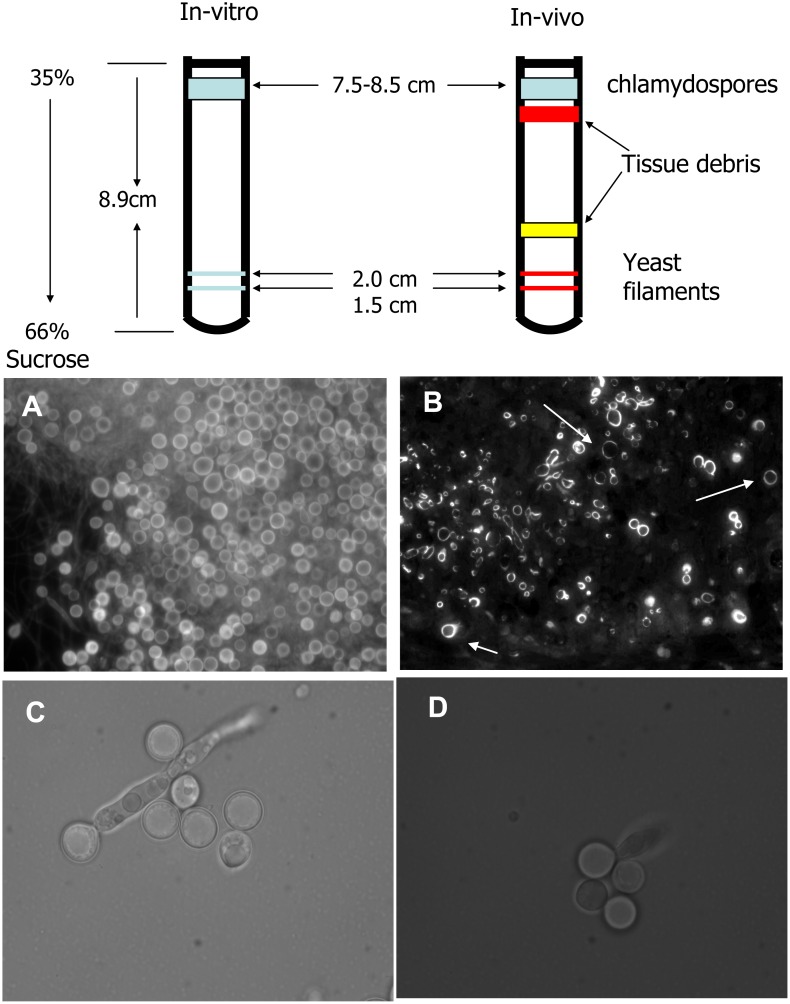
Purification of chlamydospores by sucrose density gradient. Top panel: Schematic diagram showing sedimentation pattern for chlamydospores, yeast, and filamentous *C*. *albicans* after ultracentrifugation on a sucrose density gradient. Bottom panel: A. Gradient purified chlamydospores from *in vitro* cultures imaged under fluorescence microscopy after staining with Calcofluor White. B. Thin section of infected mouse kidney 3 days PI with *C*. *albicans* and stained with Calcofluor White (*in vivo*). C &D Unstained chlamydospores examined under phase contrast microscopy at 1000X (C, *in vitro*; D, *in vivo*). A and C, purified chlamydospores from *C*. *albicans* A72 grown in cornmeal agar; B and D, harvested from *C*. *albicans* A72 infected kidneys 102 h PI.

We isolated these large cells using isopycnic sucrose density gradient centrifugation. Chlamydospore morphology was confirmed by Calcofluor White staining after purification. On 35 to 66% (w/w) linear gradients yeasts, hyphae, and pseudohyphae banded at ca. 1.28–1.29 g/cc, whereas the chlamydospores banded at ca. 1.17 g/cc (Fig 2, top panel). This lower density for chlamydospores is consistent with the presence of refractile lipid granules as observed by phase contrast microscopy as well as the high lipid content (≥ 20%) reported by Jansons and Nickerson [59] and the sucrose flotation purification described for chlamydospores by Fabry et al [17]. Without sonication the chlamydospores remained attached to hyphae and banded at an intermediate density of ca. 1.22–1.24 g/cc. Chlamydospores produced *in vitro* on cornmeal agar were identical with those formed *in vivo* three days PI in mouse kidneys with regard to their banding position on sucrose density gradients (Fig 2). Purified *in vitro* chlamydospores and thin histological sections of *Candida* infected kidneys were stained with Calcofluor White and observed by fluorescence microcopy (Fig 2A and 2B), while *in vitro* and *in vivo* purified unstained chlamydospores were examined by phase contrast microscopy at 1000X (Fig 2C and 2D). When compared with the *in vitro* cells, *Candida* infected kidney sections stained using Calcofluor White showed cells with the expected size of chlamydospores (Fig 2B). Critically, the *in vitro* and *in vivo* chlamydospores banded at the same density (1.17 g/cc, Fig 2, top panel) and had the same diameter (8.0 ±1 μm, Fig 2C and 2D). These properties indicate that the chlamydospore-like structures observed in kidney sections in the mouse model of disseminated candidiasis are indeed chlamydospores.

### Chlamydospore mutant phenotypes in a BWP17 background

**Fig 3:**
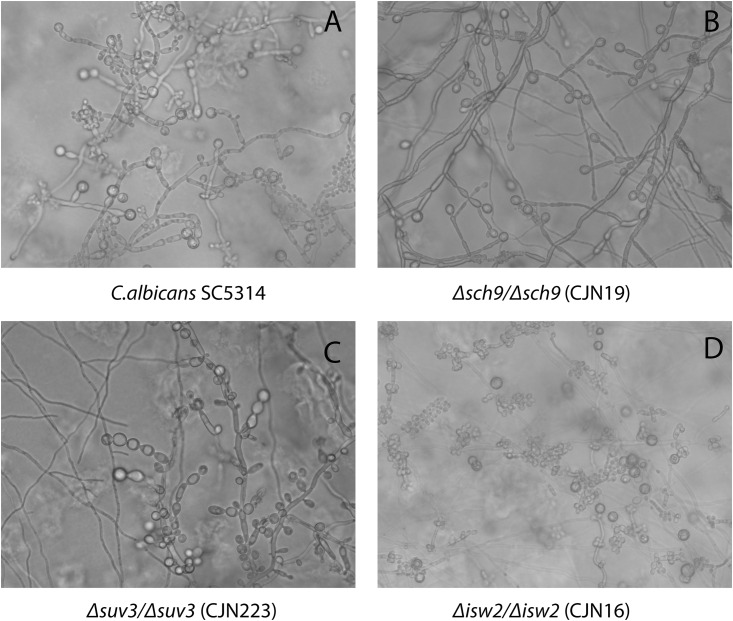
Morphology and timing of chlamydospore formation in WT *C*. *albicans* and CJN19, CJN223 and CJN16 strains grown on corn meal agar. **(A)** *C*. *albicans* SC5314 (10 days); **(B)** *C*. *albicans* CJN19 (*Δsch9/Δsch9*, 25 days) **(C)** *C*. *albicans* CJN223 (*Δsuv3/Δsuv3*, 25 days) and **(D)** *C*. *albicans* CJN16 (*Δisw2/Δisw2*, 35 days). Imaging was performed *in situ* in corn meal agar plates under bright field using an AMG EVOSfl Digital Inverted Microscope.

We reexamined the chlamydospore null mutants *Δisw2/Δisw2* (CJN16), *Δsch9/Δsch9* (CJN19) and *Δsuv3/Δsuv3* (CJN223) originally identified by Nobile et al [19]. The mutants were grown under microaerophilic conditions on corn meal agar for four weeks, and WT SC5314 and BWP17 strains were included for comparison. During the first three weeks, in agreement with previous findings [19], none of the mutants produced chlamydospores. However, prolonged incubation up to four weeks showed some chlamydospores in both *Δsch9/Δsch9* and *Δsuv3/Δsuv3* mutants, which structurally resembled the chlamydospores formed by WT SC5314 (Fig 3A versus 3B & 3C). More interestingly, the *Δisw2/Δisw2* (CJN16) mutant also showed late induction of chlamydospores during the fifth week with the unusual feature that the *Δisw2/Δisw2* mutant chlamydospores were located laterally, attached directly to the hyphae, and apparently without the usual suspensor cells (Fig 3D). For this reason, we chose to study further the function of *ISW2* in a virulent background.

### *ISW2* deletion altered *in vitro* chlamydospore formation

**Fig 4:**
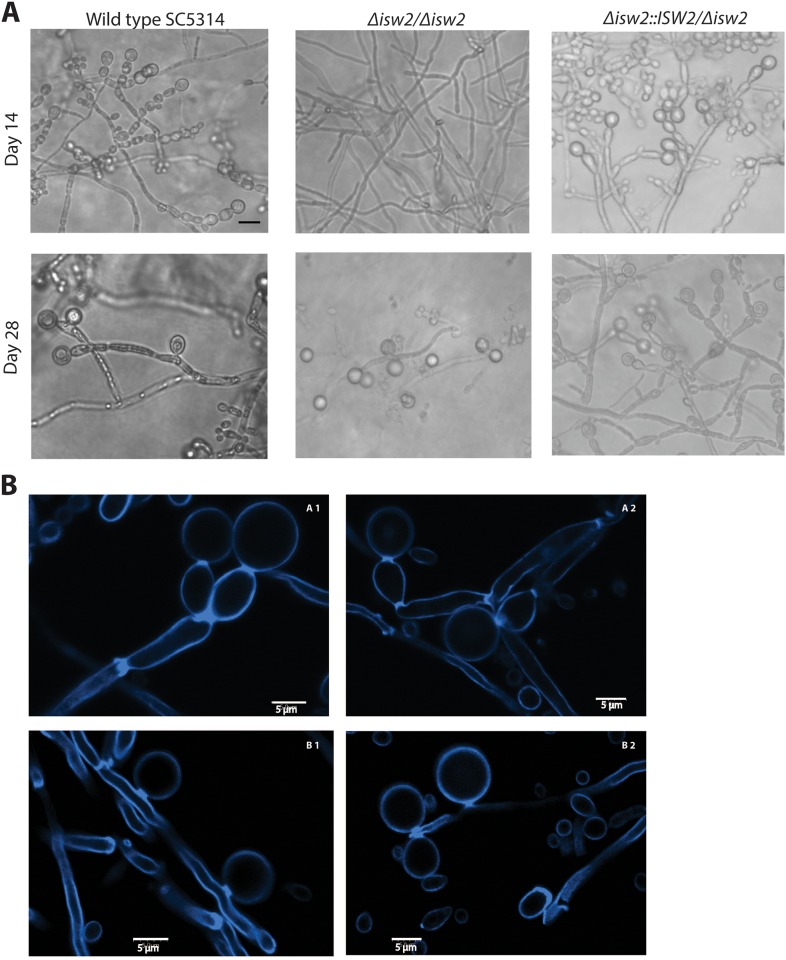
Late induction of chlamydospores lacking suspensor cells in *Δisw2/Δisw2* mutant strain compared to its parent strain. **(A)** Time course of chlamydospore development over six weeks to verify a developmental pattern of chlamydospores lacking suspensor cells in DRL6 (*Δisw2/Δisw2)* strain. Images captured at 2 and 4 weeks are shown. The WT SC5314 and *ISW2* complemented DRL7 strains initiated chlamydospore formation within 5 to 10 days on corn meal agar supplemented with 1% Tween 80. In contrast, the DRL6 (*Δisw2/Δisw2*) strain had no visible chlamydospores by day 14 but did produce lateral chlamydospores without suspensor cells after 4 to 5 weeks of incubation. **(B)** Micrographs of Calcofluor White-stained chlamydospores from *C*. *albicans* SC5314 (A1, A2) and the DRL6 (*Δisw2/Δisw2*) strain (B1, B2) captured after 2 and 4 weeks of incubation, respectively, with an Olympus FV500 Inverted (Olympus IX-81) Confocal Microscope. The average size of the WT chlamydospores formed on suspensor cells are 8.32 ± 0.94 (SD) μm, whereas the chlamydospores lacking suspensor cells are 7.88 ± 1.12 (SD) μm; n = 50. The images were analyzed with ImageJ (NIH) software for diameter measurement.

The chlamydospore-null mutants described by Nobile et al are insertion mutants derived from auxotrophic strain BWP17 (*ura3*::*imm434/ura3*::*imm434* *iro1/iro1*::*imm434 his1*::*hisG/his1*::*hisG arg4/arg4)* [19]. Some *URA3* blasted gene deletion strains are avirulent in mouse models due to positional effects on expression of reintroduced URA3 or the unintended deletion of *IRO1*, a virulence factor tightly linked to *URA3* gene [60–63]. To avoid such potential artifacts in subsequent mouse virulence studies, we constructed a *Δisw2/Δisw2* mutant in a prototropic background using the method of Reuss et al. [49, 50] as we previously reported [30, 34]. Consistent with the report by Nobile et al [19], our *ISW2* deleted (DRL6) strain constructed in the fully virulent SC5314 WT background did not exhibit chlamydospore production up to 21 days following induction (Fig 4A), while the reconstituted strain formed mature chlamydospores within 14 days (Fig 4A). However, during the fourth week of incubation, the DRL6 strain formed laterally located, chlamydospore-like thick walled cells, connected to the hyphae without any intervening suspensor cells (Fig 4A and 4B). These putative chlamydospores were harvested and purified by sucrose density gradients, where they banded at the same low density as WT chlamydospores, a feature characteristic of their high lipid content [59]. Microscopy of these putative chlamydospores revealed thick-walled spheres with diameters of 7.9±1 μm as expected for chlamydospores. Furthermore, the cells were readily stained with low concentrations of Calcofluor White (Fig 4B), which revealed that the lateral chlamydospores made by DRL6 were directly connected to the hyphae by spore-hypha septa (Fig 4B). These septa are important because they indicate a regulated sporulation process rather than merely a cytoplasmic burst through a weakened cell wall.

Notably, we have also observed the rare appearance of chlamydospores lacking suspensor cells in aged WT SC5314 cultures (Fig 4A). We also compared several previously described mutants with abnormal chlamydospore phenotypes [19, 64, 65] for longer incubation periods to verify that suspensor cells are specifically regulated by *ISW2* gene. Among those chlamydospore-defective mutants, DAY25 (*rim101Δ/rim101Δ)* [19], hyphal growth defective BH1P1 (BMH1/*bmh1Δ*), and UdR142C (*bmh1Δ/bmh1Δ)* [64, 65] showed a similar abundance of chlamydospore formation as the WT strains with sporadic occurrence of chlamydospores without suspensor cells by the end of five weeks of incubation. Due to the presence of chlamydospores, with and without suspensor cells and their small numbers with the latter phenotype, these observations are not comparable to that of *ISW2* deletion strains of DRL6 or CJN19. Therefore, *ISW2* may play a regulatory role in formation of suspensor cells for the chlamydospores and the onset of chlamydospore formation.

### Effect of *ISW2* deletion on filamentation, cell cycle progression, and stress responses

**Fig 5:**
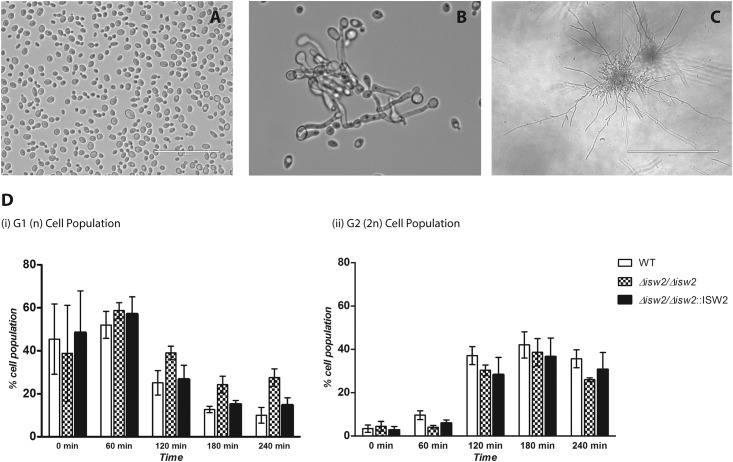
Filamentation morphologies and cell cycle progression is unaltered by *ISW2* deletion. **(A)** Yeast cell morphology after overnight growth in YPD at 30°C. **(B)** Hyphal formation after overnight growth in YPD at 37°C. **(C)** Vigorous filamentation in embedded GPP agar after 16 hrs at 37°C. **(D)** Histograms from flow cytometry analysis of cell cycle for each cell population (G1 and G2) percentage from three biological replicates. Representative flow cytometry plots shows that cell cycle progression is similar to that of WT strain. G1 synchronized cells were analyzed via a time course with PI staining as described under Materials and Methods.

Based on previous observations that filamentation is a prerequisite for chlamydospore formation [14], we examined the filamentous growth capabilities of the DRL6 strain under two standard filament inducing conditions (YPD liquid and GPP embedded agar, both at 37°C) and found no differences compared with WT *C*. *albicans* (Fig 5A–5C). Furthermore, the DRL6 strain readily formed hyphae on corn meal agar that were indistinguishable from WT hyphae.

We examined cell cycle progression profiles of the DRL6 strain compared to its WT parent. Propidium iodide-stained cells were examined via flow cytometry and by quantification of the cell numbers (Fig 5D). No differences in cell cycle phase distribution (G1 vs. G2) were observed between DRL6 and SC5314 strains under the standard growth conditions.

*C*. *albicans* confronts diverse stresses during host adaptation and pathogenicity. Because many genes identified in chlamydospore formation are also involved in stress responses [15, 19], we carried out spot dilution assays to examine the stress tolerance of the DRL6 strain towards H_2_O_2_ (up to 4.0 mM), sodium chloride (up to 1.5 M), sorbitol (up to 2.0 M), and farnesol (up to 200 μM). None of these stress inducers differentially affected the growth rate of DRL6 versus WT SC5314 strains at the concentrations tested (data not shown). This absence of hypersensitivity to oxidative stress is consistent with previous observations for the CJN16 strain [19]. Taken together, the absence of defects in filamentation, cell cycle progression, and stress responses in DRL6 mutant makes it more likely that *ISW2* has a specific function in the timing of chlamydospore formation via the presence or absence of suspensor cells.

### *ISW2* deletion promotes chlamydospore formation in Staib agar

**Fig 6:**
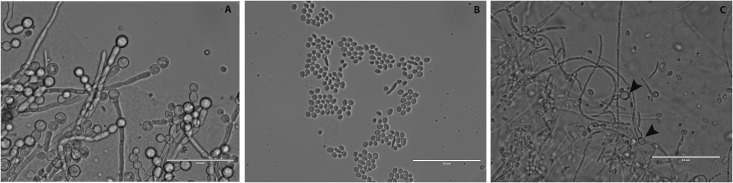
DRL6 induces chlamydospores in Staib agar. **(A)** Abundant chlamydospore formation by *C*. *dubliniensis* Wü284. **(B)** Poor filamentation and no detectable chlamydospores by *C*. *albicans* SC5314. **(C)** Occasionally visible chlamydospore formation by DRL6 (*Δisw2/Δisw2*) strain in Staib agar as indicated by the black arrow heads. Plates were grown at room temperature in the dark for up to 7 days, and representative pictures are from at least 3 independent experiments.

Chlamydospore formation in Staib agar is diagnostic for *C*. *dubliniensis* in that *C*. *albicans* is unable to induce chlamydospores in the same medium [47]. Later, Staib and Morschhäuser showed that down regulation of *NRG1* in *C*. *dubliniensis* allows chlamydospore formation in Staib agar and, as a corollary, that *CaNRG1* acts as a repressor of this ability. Consequently, the *nrg1Δ/nrg1Δ* mutant of *C*. *albicans* produced chlamydospores in Staib medium [21, 23]. In *C*. *albicans*, the *NRG1* gene encodes a transcriptional repressor of filamentation that acts in part synergistically with *TUP1* gene, a transcriptional corepressor. Therefore, we examined the growth behavior of DRL6 (*Δisw2/Δisw2*) strain on Staib agar in comparison to *C*. *dubliniensis* and WT SC5314. Within a week of incubation, *C*. *dubliniensis* produced filaments and chlamydospores (Fig 6A) whereas SC5314 as expected, failed to produce filaments or chlamydospores (Fig 6B). Surprisingly, the DRL6 strain showed moderate filamentation and sporadic occurrence of some conventional prototype chlamydospores (i.e. with suspensor cells) on Staib agar (Fig 6C). We have never observed chlamydospores with suspensor cells for the DRL6 strain on corn meal agar. The *Δnrg1/Δnrg1* mutant was reported to produce chlamydospores occasionally on corn meal agar[23]. Thus, the appearance of chlamydospores with suspensor cells on Staib agar is intriguing because it shows that suspensor cell production is nutritionally regulated.

### *ISW2* deletion reduces virulence and alters the inflammatory immune response in the mouse model of disseminated candidiasis

**Fig 7:**
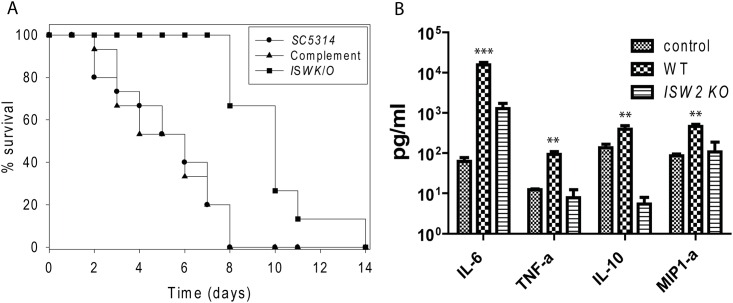
Deletion of *ISW2* prolongs survival in the mouse model of systemic candidiasis and alters host cytokine and chemokine expression. **(A)** Effect of *ISW2* deletion on mouse survival following intravenous *C*. *albicans* injection. Survival of mice injected with WT *C*. *albicans* SC5314 (●), the null mutant DRL6 (*Δisw2/Δisw2*) (■) and single copy reconstituted DRL7 (▲). Gehan-Breslow-Wilcoxon test hazard ratio estimates indicated 3.4-times greater lethality for WT infection compared to infection by the DRL6 strain. (n = 15; p<0.001, uninfected control = 6, data not shown). **(B)** Effects of Isw2p expression in *C*. *albicans* on host serum cytokine and chemokine induction after infection. Serum levels of the indicated cytokines (IL-6, TNF-α, IL-10, and MIP1α) were assessed at day 2 PI for mice infected with WT (checkerboard) or DRL6 (*Δisw2/Δisw2*) strain (horizontal lines) and statistically compared with both uninfected control and mutant. Control (crosshatch) values at day 0 are mean values determined for sera from five uninfected mice. Quantitative data represent mean ± SEMD. ** = p< 0.01; *** = p< 0.001.

The well-established mouse model of disseminated candidiasis was used to compare virulence among the WT, DRL6 (*Δisw2/Δisw2*), and DRL7 (*Δisw2*::*ISW2/Δisw2*) strains. Mice infected with the DRL6 strain had a significantly higher survival rate compared with mice infected with the WT SC5314 strain (n = 15, p<0.001, Fig 7A). A control group of mice that received sterile non-pyrogenic saline i.v. did not show any mortality. The Gehan-Breslow-Wilcoxon test hazard ratio estimates indicated 3.4-times greater lethality for WT infection compared to infection by the DRL6 strain. Complementation of one allele in the DRL7 strain restored virulence to the level of the WT parent strain and significantly increased virulence compared with the DRL6 strain (Fig 7A, p<0.001).

We screened a panel of cytokines and chemokines at day 2 PI to assess the innate immune response to infection. WT infected mice had significantly higher serum levels of IL-6, TNF-α, IL-10 and MIP1-α compared with both the mutant infected mice and the uninfected control group (Fig 7B). Deletion of ISW2 did not significantly alter the responses to infection of serum cytokines IL-17, IL-1β, GM-CSF and the chemokines MCP-1 and RANTES relative to that observed in infected WT mice (Figure A in S1 File).

### Absence of chlamydospores in kidneys infected with the DRL6 mutant

**Fig 8:**
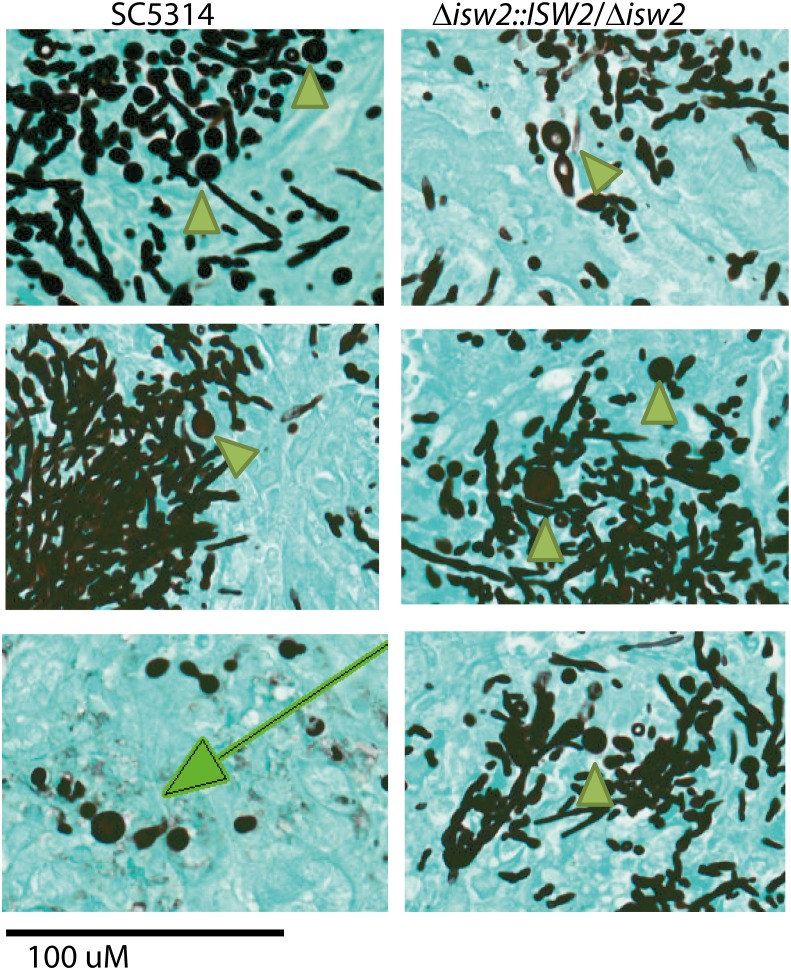
Isw2p expression induces chlamydospore formation in kidneys of mice with disseminated candidiasis. Representative GMS stains of kidney sections dissected from mice infected with WT, and reconstituted DRL7 strains. Arrowheads indicate representative chlamydospores in histological sections of infected mouse kidneys at 3–6 days PI. Large arrow in left-lower corner image indicates a solitary chlamydospore in a resolving lesion in mouse kidney cortex at 8 days PI. Scale bar, 100 μm.

We next examined the *in vivo* formation of chlamydospores by the DRL6 strain compared to WT SC5314. We examined infected mouse kidneys harvested from the survival assays (Fig 7A) described in the previous section to determine whether DRL6 strain lost its ability to form chlamydospore-like structures *in vivo*. Infected kidneys were examined at the subacute phase (day 3–6) when the kidney cortex is predominantly colonized and chlamydospore-like structures are first observed [34]. Thorough examination of representative sections showed no chlamydospore-like structures in kidneys from mice infected using the DRL6 strain (Figure B in S1 File). In contrast, we consistently observed chlamydospore-like cells in kidneys from mice infected with SC5314 or the *ISW2*-complemented DRL7 strains (Fig 8). Therefore, the large cells (arrow heads) noted in GMS stained sections from WT and DRL7 strains are most likely chlamydospores, as indicated by the 100 μm size bar positioned at the bottom of the panel (Fig 8). A few solitary chlamydospores, indicated by the arrow in a representative section at day 8 PI, were visible in SC5314 WT infected kidney matrix in the resolution phase. Furthermore, we did not observe overwhelming kidney colonization by DRL6 compared with the characteristic phenotypes of SC5314 and DRL7 strains (Figure B in S1 File), consistent with the lower virulence of the DRL6 strain (Fig 7A). Otherwise, the DRL6 strain did not deviate from the kidney pathogenesis pattern of WT [30, 34, 35] and progressed to medullary colonization at the chronic stage of kidney infection (Figure B in S1 File).

### *CSP1* and *CSP2* gene expression *in vivo* and *in vitro*

**Fig 9:**
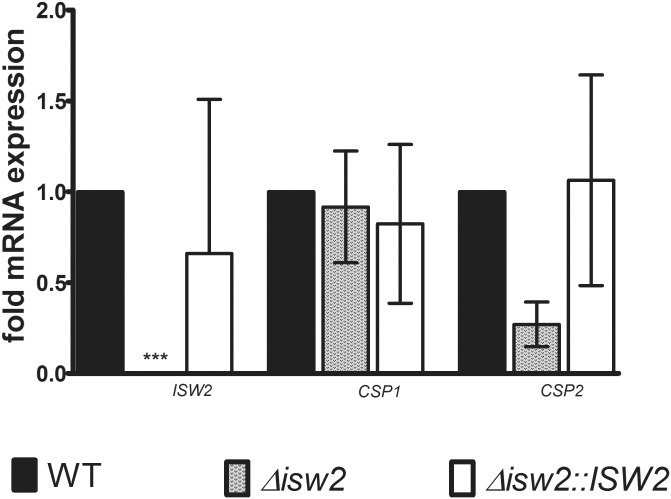
Expression of the chlamydospore-specific genes *CSP1* and *CSP2* during *in vivo* growth of *C*. *albicans*. qRT-PCR analysis of total RNA extracted from infected mouse kidneys harvested at 3 days PI showing that *CSP1* and *CSP2* were expressed during infection *in vivo* by all three strains. Expression is presented normalized to 1 for the WT strain, where mean Ct values were 24.3 for *ISW2*, 35.59 for *CSP1*, and 35.61 for *CSP2*. Results represent mean ± SD from three biological replicates. *** = p< 0.001.

We investigated expression of two recently reported chlamydospore-associated markers, *CSP1* (orf19.3512) and *CSP2* (orf19.4170) [24], *in vivo* in infected kidneys at day 3 PI (Fig 9). We examined *ISW2*, *CSP1* and *CSP2* gene expression in DRL6 and DRL7 strain infected kidneys compared with SC5314 WT infected kidneys. Three days coincides with the onset of mortality for WT *C*. *albicans* (Fig 7A) and our current and previous observations of chlamydospore appearance in infected kidneys. Interestingly, neither *CSP1* nor *CSP2* expression was affected by *ISW2* deletion (p < 0.05) under these *in vivo* conditions (Fig 9).

In parallel, we compared the expression of *CSP1* and *CSP2* in cells grown in YPD and corn meal broth. Basal expression of *CSP1* in YPD was not significantly affected by deletion of *ISW2*, whereas *CSP2* expression decreased in the mutant and was restored in the complemented strain (Figure C panel B in S1 File). Growth of the *ISW2* deletion mutant in cornmeal medium for five days in the dark resulted in significant induction of *CSP2* mRNA relative to that in YPD or basal expression in corn meal medium (Figure C panel D, F in S1 File). In the same strain, *CSP1* showed significant induction relative to basal expression in cornmeal medium on day 5 but not relative to that in YPD (Figure C panel C, E in S1 File). No significant changes in *CSP1* or *CSP2* expression were observed in corn meal medium for WT SC5314 or the reconstituted strain. These data suggest that *ISW2* is not necessary for induction of *CSP1* or *CSP2* expression. The previously reported strong upregulation of *CSP1* and *CSP2* during chlamydospore induction may depend on undefined factors in Staib or rice extract media that are not optimal in corn meal medium.

## Discussion

*In vivo* chlamydospore formation during *C*. *albicans* infections and their role in virulence have been poorly studied to date. Here we document the formation of chlamydospores in *Candida*-infected mouse kidneys and reduced virulence of a *Δisw2/Δisw2* strain in a mouse model of disseminated candidiasis that coincides with an absence of chlamydospores in infected kidneys. We also identify *ISW2* as a gene that is not required for chlamydospore formation but rather determines the timing and morphology of chlamydospore development. In an *ISW2* null background, chlamydospores form laterally from the hyphae without an apparent suspensor cell, which challenges the dogma that suspensor cells are necessary for chlamydospore formation. Although the *ISW2* deleted strain is defective in producing suspensor cells, *ISW2* is not necessary for filamentation, cell cycle progression, or induction of the chlamydospore markers *CSP1* and *CSP2* in corn meal medium. Delayed chlamydospore induction upon *ISW2* deletion under laboratory conditions is consistent with the absence of chlamydospores in kidneys infected with this strain at day 3 PI.

The ability to produce chlamydospores *in vitro* under inducing conditions is nearly universal among clinical isolates of *C*. *albicans* [66]. Given that yeast-mycelia morphogenesis is fundamental to the dissemination and virulence of *C*. *albicans*, we reasoned that chlamydospore morphogenesis may also play some pathophysiological role. Defining a role of chlamydospores in *C*. *albicans* pathogenesis requires careful analysis of clinical samples to exclude chlamydospores associated with secondary mycotic infections. Chlamydospores resemble large blastoconidia of *C*. *albicans* [67, 68] and a variety of morphological forms of the common fungal pathogens *Cryptococcus neoformans*, *Blastomyces dermatitidis*, and *Paracoccidioides brasiliensis* [67]. Such confusion in diagnosis could be prevented by identifying unique morphological, physiological and molecular features of *C*. *albicans* chlamydospores. Their characteristically large size compared to vegetative cells, staining characteristics including their double walled spherical morphology, distinctive buoyant density, the presence of lipid granules, gene expression, and association with suspensor cells confirm the chlamydospore formation by *C*. *albicans in vitro* as well as *in vivo*.

Chlamydospore-like structures were previously reported in human clinical specimens from skin, brain, gastrointestinal tract, endocardium and kidneys [26, 69–71]. A clinical case report noted giant forms of *C*. *albicans* that resemble chlamydospores in multiple organs including the lungs and pancreas [67]. Those authors suggested that treatment with antimicrobial agents could have induced formation of these large structures. Chlamydospore-like structures were also observed in the gastrointestinal tract of infected immunocompromised mice [25]. Candidiasis patients were found to produce antibodies reactive with chlamydospores [72].

Most of these early reports of *in vivo* chlamydospores predate the identification of *C*. *dubliniensis* in 1995 as a new *Candida* species that forms chlamydospores more readily than does *C*. *albicans* [73]. Therefore, these early clinical observations cannot be attributed to *C*. *albicans* rather than *C*. *dubliniensis*. We have also observed variation within *C*. *albicans* in that strains A72 and 10231 form chlamydospores more readily and abundantly than SC5314 (unpublished observation).

The expression of *CSP1* and *CSP2* in the DRL6 (*Δisw2/Δisw2)* mutant *in vitro* and in infected kidneys is consistent with a RNASeq analysis of a *C*. *albicans Δnrg1* mutant or *C*. *dubliniensis* grown in Staib medium, which did not find significant changes in *ISW2* or other genes important for chlamydospore formation including *SUV3*, and *SCH9* relative to WT *C*. *albicans* [19, 24]. Therefore, *ISW2* expression is not dependent on *CSP1* or *CSP2* expression levels under the specific growth conditions studied. Conversely, deletion of *ISW2* decreased the basal expression of *CSP2* mRNA in YPD but did not alter transient induction of *CSP1* or *CSP2* gene expression in cornmeal medium. Although Csp1p and Csp2p protein expression is specific for chlamydospores, the mRNAs encoding these proteins are also expressing basally in YPD medium where no chlamydospores are present [24], and we have confirmed this result in our strains. Therefore, regulation of Csp1p and Csp2p protein levels may occur post-transcriptionally, and background expression of their mRNAs by yeast cells precludes any conclusion that the CSP1 or CSP2 mRNA expression we observed in infected kidneys is associated with chlamydospores.

The reduced virulence of the *ISW2* null mutant DRL6 in a mouse model of disseminated candidiasis was associated at day 2 PI with decreased up-regulation of IL-6, TNF-α, MIP1-α, and IL-10, cytokines and chemokines that are known to play a role in innate host defense against *Candida in vivo* [58, 74, 75]. These differences may simply reflect the lower fungal load associated with the mutant *Candida* strain, but further investigation into the effects of *ISW2* on the host immune response is warranted. The persistence of chlamydospores we observed in resolving *Candida* lesions in infected mouse kidney cortex suggests a potential role in resistance to host immunity.

*C*. *albicans ISW2* is not well-studied, but potential functions can be predicted based on studies of the *S*. *cerevisiae* ortholog. A BLAST (bl2seq) search of the amino acid sequence of Isw2p of *C*. *albicans* (orf19.7401) versus Isw2p of *S*. *cerevisiae* (YOR304W) using the NCBI BLASTP tool identified 61% identity and 77% similarity between the two sequences. The yeast replicative life span was extended robustly upon *ISW2* deletion in *S*. *cerevisiae*, which was accompanied by derepression of a cohort of stress responses genes [76]. This negative regulation mechanism was also observed in *C*. *elegans* as well as in other complex eukaryotes [41, 76], providing further evidence for a highly conserved function of the ISWI subfamily. However, we did not observe enhanced sensitivity to osmotic or oxidative stress in the DRL6 strain compared with the WT SC5314 parent, and therefore delayed chlamydospore formation could not be solely attributed to differential stress responses in the mutant strain [76]. The question remains whether suspensor cell and/or chlamydospore formation could be governed by a nutritional stress rather than an oxidative or osmotic stress.

The mechanism by which the *ISW2* deletion induces regular chlamydospores in Staib agar needs further investigation because *C*. *albicans* does not induce chlamydospores in Staib agar except with a *Δnrg1/Δnrg1* mutant. One possibility is downregulation of *NRG1* in the *ISW2* deleted DRL6 strain. *In vitro* chlamydospore formation is typically induced in unusual growth media with complex carbohydrate sources. Nutrient depletion conditions can signal stress responses via the TOR signaling pathway and the *ISW2*-regulated pathway [76]. *SCH9* encodes a nutrient-responsive protein kinase that acts in parallel to TOR to regulate replicative life span [77]. *C*. *albicans Δsch9/ Δsch9* (CJN19) also exhibits defects in its chlamydospore formation kinetics (This study and [19]). Since chlamydospore formation occurs upon prolonged incubation, which results in accumulation of aged cells, and is limited to nutritionally-poor media such as corn meal agar or rice extract agar, such caloric restriction conditions may regulate unknown chlamydospore-inducing genes.

Our studies identify *ISW2* as a regulator of the *C*. *albicans* suspensor cells associated with chlamydospore formation. Our finding of chlamydospore formation in the absence of suspensor cells could account for the previously reported lateral chlamydospores in WT isolates [78]. Further physical and molecular characterization is needed to confirm those morphological observations. Preliminary morphological evidence for direct formation of chlamydospores from yeast cells has also been reported [79]. However, the infrequent occurrence of lateral chlamydospores in aged yeast cultures needs further examination. Many details of the molecular function of Isw2p in *C*. *albicans* remain unclear. Identifying infection-associated genes regulated by Isw2p will be paramount to understanding the developmental and regulatory steps governing chlamydospore formation and their contribution to pathogenesis and modulation of host responses during systemic and mucosal *C*. *albicans* infections.

## Supporting Information

S1 File **Table. Primers used in this study.** Underlined segment indicate the custom restriction sites inserted for constructing pSFS2A*ISW2* and p*ISW2COMP*. **Figure A. Host serum cytokine and chemokine responses after infection show no significant dependence on ISW2.** The cytokines IL-17, IL-1β, GM-CSF as well as the chemokines MCP-1, and RANTES did not exhibit significant differences at day 2 PI for mice infected with WT (checkerboard) or DRL6 (*Δisw2/Δisw2*) strain (horizontal lines). Control (crosshatch) values at day 0 are mean values determined for sera from five uninfected mice. Quantitative data represent mean ± SEM. **Figure B. Histopathological observations in kidney sections of mice infected with WT, DRL6 (*Δisw2/Δisw2*) mutant and reconstituted DRL7 strains.** Representative GMS stains of kidney sections dissected from mice infected with *ISW2* deleted DRL6, showing no chlamydospores in comparison to the wild type and *ISW2* complemented strain where arrowheads indicate representative chlamydospores. **Figure C. Expression of the chlamydospore-specific genes *CSP1* and *CSP2* during *in vitro* growth of *C*. *albicans*.** *C*. *albicans* SC5314, DRL6, and DRL7 strains were grown *in vitro* in corn meal or YPD broth media, and total RNA was isolated at days 1, 2, 3 and 5. Basal expression in YPD is shown in panels A and B. The fold expression of *CSP1* and *CSP2* in cornmeal medium were normalized to respective gene expression on day 1 under non-inducing (C, D) and inducing conditions respectively (E, F). qRT-PCR analysis showed that *ISW2* deletion significantly affected the expression of the chlamydospore-specific markers, *CSP1* (A) and *CSP2* only on day 5 (B). Results represent mean ± SD from three biological replicates. Quantitative data represent mean ± SD. * = p<0.05; ** = p< 0.01; *** = p< 0.001.
(PDF)
