# Supplementary material for: Candida albicans ISW2 Regulates Chlamydospore Suspensor Cell Formation and Virulence In Vivo in a Mouse Model of Disseminated Candidiasis
Source: PLoS One. 2016 Oct 11;11(10):e0164449. doi: 10.1371/journal.pone.0164449 (PMC5058487; doi:10.1371/journal.pone.0164449)
Supplement: S1 File — Table. Primers used in this study. Underlined segment indicate the custom restriction sites inserted for constructing pSFS2AISW2 and pISW2COMP. Figure A. Host serum cytokine and chemokine responses after infection show no significant dependence on ISW2. The cytokines IL-17, IL-1β, GM-CSF as well as the chemokines MCP-1, and RANTES did not exhibit significant differences at day 2 PI for mice infected with WT (checkerboard) or DRL6 (Δisw2/Δisw2) strain (horizontal lines). Control (crosshatch) values at day 0 are mean values determined for sera from five uninfected mice. Quantitative data represent mean ± SEM. Figure B. Histopathological observations in kidney sections of mice infected with WT, DRL6 (Δisw2/Δisw2) mutant and reconstituted DRL7 strains. Representative GMS stains of kidney sections dissected from mice infected with ISW2 deleted DRL6, showing no chlamydospores in comparison to the wild type and ISW2 complemented strain where arrowheads indicate representative chlamydospores. Figure C. Expression of the chlamydospore-specific genes CSP1 and CSP2 during in vitro growth of C. albicans. C. albicans SC5314, DRL6, and DRL7 strains were grown in vitro in corn meal or YPD broth media, and total RNA was isolated at days 1, 2, 3 and 5. Basal expression in YPD is shown in panels A and B. The fold expression of CSP1 and CSP2 in cornmeal medium were normalized to respective gene expression on day 1 under non-inducing (C, D) and inducing conditions respectively (E, F). qRT-PCR analysis showed that ISW2 deletion significantly affected the expression of the chlamydospore-specific markers, CSP1 (A) and CSP2 only on day 5 (B). Results represent mean ± SD from three biological replicates. Quantitative data represent mean ± SD. * = p<0.05; ** = p< 0.01; *** = p< 0.001. (PDF) [file pone.0164449.s001.pdf]

S1 Table

Primer Name	Sequence (5' → 3')	Source
ISW2upleft	gtgctagaGgGcCctatcaactttgg	This study
ISW2upright	gatctCGAgttgaaattcttgatgtc	This study
ISW2downleft	*gaagttaCcgCGGatgaacagacagt*	This study
ISW2downright	taggaaacatgaGCtCagtgacatta	This study
ISW2compleft	atcGGGccCtaaataaagcacaacaa	This study
ISW2compright	gctaagctCGaGcaaaagctagaagtt	This study
Ca3512_fw1	ACACCACTGCAAGTATCCATATTGTGA	Palige et al [23]
Ca3512_rev1	ATCTTGTATAACCCTTTGTCGTCAAC	Palige et al [23]
Ca4170_fw1	GCTACTGGTGAAATTGTTGCTAATC	Palige et al [23]
Ca4170_rev1	TCATCATCACAGTCATCGCTATC	Palige et al [23]
P26	AACATTTGTGGTGAACAAGGATG	Pendrak et al [54]
P90	TGATGGTGTTACTCACGTTGTTCC	Pendrak et al [54]
CDC36	GAGCGTCCAGTATAAATCCACCAC	Pendrak et al [54]
CDC36	TCAAGACGGGCTCCACATTACTAT	Pendrak et al [54]

S1 Figure A

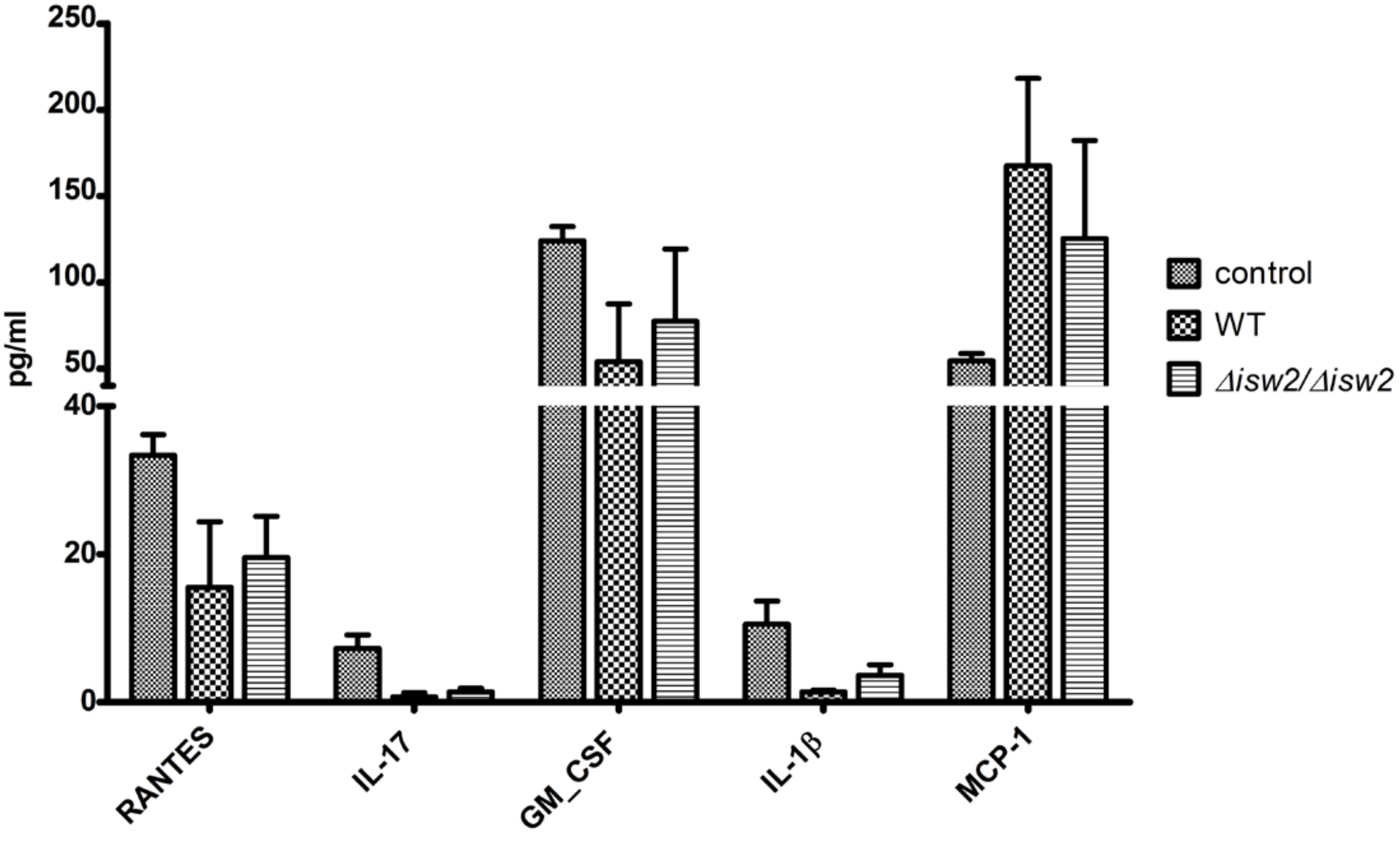

250
200
150
100
50
40
20
0
pg/ml
RANTES
IL-17
GM_CSF
IL-1β
MCP-1
control
WT
Δisw2/Δisw2

S1 Figure B

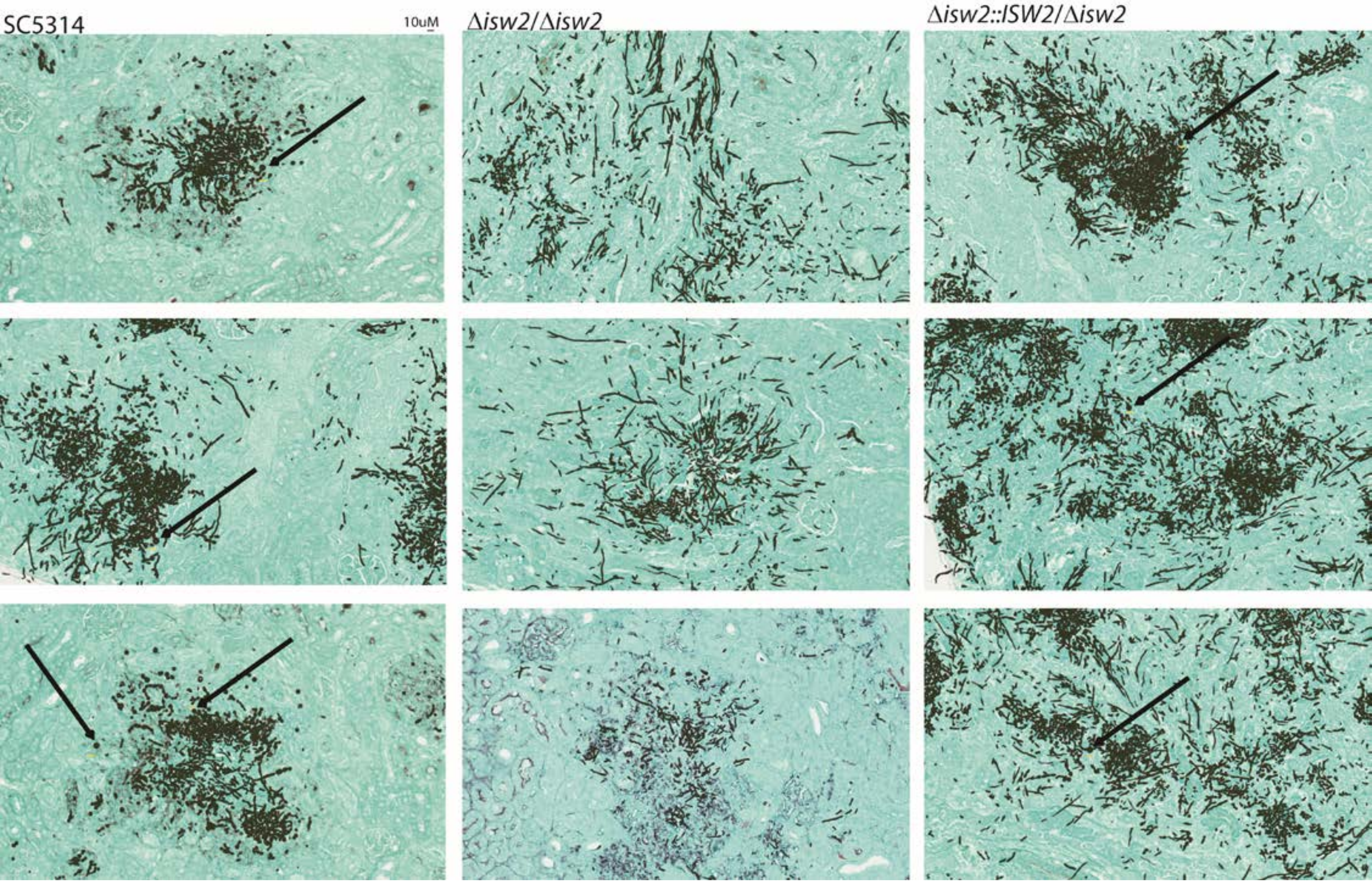

S1 Figure C

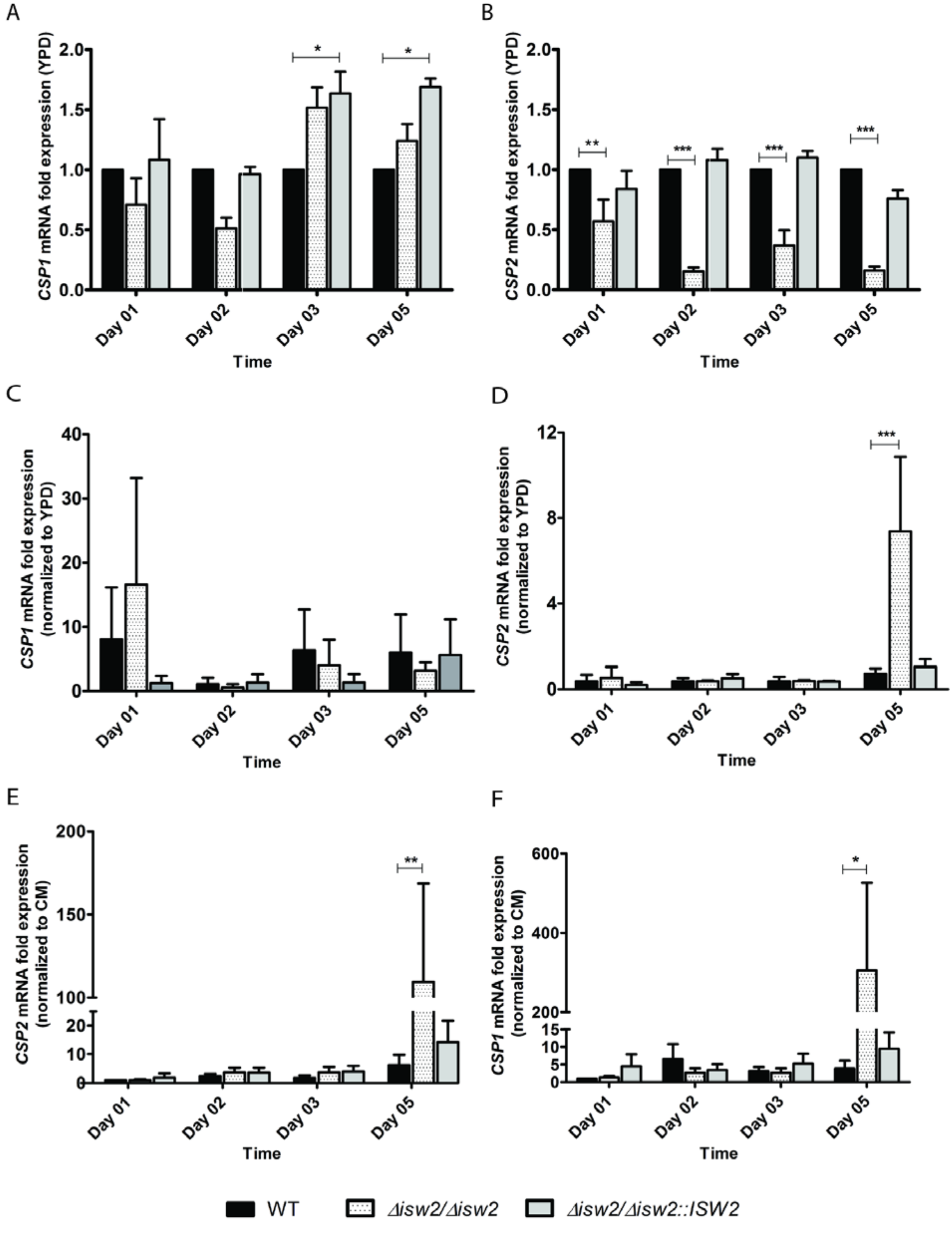
A
CSP1 mRNA fold expression (YPD)
B
CSP2 mRNA fold expression (YPD)
C
CSP1 mRNA fold expression (normalized to YPD)
D
CSP2 mRNA fold expression (normalized to YPD)
E
CSP2 mRNA fold expression (normalized to CM)
F
CSP1 mRNA fold expression (normalized to CM)
Day 01
Day 02
Day 03
Day 05
Time
WT
Δisw2/Δisw2
Δisw2/Δisw2::ISW2
